# Use of Berlin questionnaire in comparison to polysomnography and home sleep study in patients with obstructive sleep apnea

**DOI:** 10.1186/s12931-019-1009-y

**Published:** 2019-02-22

**Authors:** Susanna S. Ng, Wilson Tam, Tat-On Chan, Kin-Wang To, Jenny Ngai, Ken K. P. Chan, Wing-Ho Yip, Rachel L. Lo, Karen Yiu, Fanny W. Ko, David S. Hui

**Affiliations:** 1 Division of Respiratory Medicine, The Chinese University of Hong Kong, Sha Tin, Hong Kong; 2 SH Ho Sleep Apnoea Management Center, The Chinese University of Hong Kong, Sha Tin, Hong Kong; 3 Alice Lee Centre for Nursing Studises, National University of Singapore, Singapore, Singapore; 4 Department of Medicine and Therapeutics, The Chinese University of Hong Kong, Prince of Wales Hospital, 30-32 Ngan Shing St, Shatin, N.T Hong Kong

**Keywords:** Obstructive sleep apnea, Questionnaire, Polysomnography, Risk, Sensitivity and specificity

## Abstract

**Background:**

Obstructive sleep apnea syndrome (OSAS) is a common disorder with significant morbidity and mortality. We aimed to evaluate the predictive accuracy of the Berlin questionnaire in patients with suspected OSAS undergoing PSG in the sleep laboratory setting against those going through the Embletta™ portable diagnostic system (Embletta PDS) at home.

**Methods:**

Patients with suspected OSAS were recruited from respiratory clinics to complete Berlin questionnaire and Epworth Sleepiness Score (ESS). Patients were randomized to undergo either home-based sleep test (group A) or hospital-based polysomnography (PSG) (group B).

**Results:**

Three hundreds and sixteen subjects with newly referred suspected OSAS were recruited and randomized into group A (*n* = 157) and group B (*n* = 159). The prevalence of moderate to severe OSAS defined as apnea-hypopnea index (AHI) ≥ 15/h was 54%. The Berlin questionnaire identified 69.7% (*n* = 99) of subjects as high risk in group A and 77.5% (*n* = 100) in group B. The sensitivity, specificity, negative predictive value (NPV), and positive predictive value (PPV) of the questionnaire to predict an AHI ≥ 15/h as diagnosed by PSG was 78, 23, 67 and 35%. When compared with Embletta PDS, the specificity and NPV increased to 48 and 63%. The area under the Receiver Operator Curve (ROC) based on PSG (AUC = 0.539, 95%CI 0.417, 0.661) and based on home Embletta (AUC = 0.712, 95%CI 0.617, 0.907).

**Conclusions:**

The questionnaire was not reliable in predicting OSAS through PSG AHI whereas there was some predictive ability in discriminating patients with OSAS from normal subjects based on home Embletta sleep test.

**Trial registration:**

The study was registered at ClinicalTrials.gov (Identifier: NCT01828216) on 10 April 2013.

## Background

Obstructive sleep apnea syndrome (OSAS) is a common disorder with prevalence rates of at least 4% among the middle-aged male Caucasians and Hong Kong (HK) Chinese populations [1–3]. It is characterized by repetitive episodes of upper airway obstruction, causing intermittent hypoxia, sleep fragmentation, disabling daytime sleepiness, impaired cognitive function and poor health status [4]. OSAS patients are at increased risks of cardiovascular morbidity and mortality including sudden death, [5, 6] in addition to being more prone to road traffic accidents [7]. Despite the availability of effective therapy such as continuous positive airway pressure (CPAP), OSAS is under-diagnosed in the general population because of limited access to sleep laboratories for nocturnal polysomnography (PSG), which is labor-intensive and currently regarded as the gold standard for confirmation of sleep apnea [8, 9]. Although the use of validated portable monitoring devices is proven to shorten waiting time and save cost, [10] the next impediment to the diagnosis is the relative lack of qualified sleep physicians. Simplified strategies with the use of questionnaires have been proposed to help predicting OSAS in the primary care setting [11, 12]. Berlin questionnaire has been developed as a tool in screening OSAS and validated in primary care [13]. It categorizes patients as either high or low risk for OSAS based on self-reports of snoring, daytime sleepiness, hypertension and obesity. However, most published studies validating its use in predicting OSAS were compared against PSG [13–15] with variable results in sensitivity and specificity. While there is a growing use of portable home monitoring in diagnosing OSAS, the performance of this questionnaire against the portable monitoring devices and PSG is needed.

## Methods

This study aimed to evaluate the accuracy of the Berlin questionnaire in patients with suspected OSAS in comparisons against PSG in the sleep laboratory setting and the Embletta™ portable diagnostic system (Embletta PDS) at home.

This study is an ancillary study of a randomized controlled trial (RCT) regarding the use of ambulatory approach versus the hospital-based approach in managing 316 clinic patients with suspected OSAS [10]. Patients with new referrals to the Respiratory Clinic, Prince of Wales Hospital, Shatin, were recruited from 25 September 2013 to 31 August 2014. OSAS was defined by apnea-hypopnea index (AHI) ≥ 5/h of sleep plus excessive daytime sleepiness or two of the following symptoms: choking or gasping during sleep, recurrent awakenings from sleep, unrefreshed sleep, daytime fatigue, and impaired concentration [16].

Patients aged 18–80 years with suspected OSAS underwent assessment at the clinic with the Epworth sleepiness score (ESS) [17] and symptoms evaluation. Patients who had ESS score > 9 or at least two OSAS symptoms as described above were invited to join the study. *Exclusion criteria* included patients with (a) unstable cardiovascular diseases (e.g. recent unstable angina, myocardial infarction, stroke within the previous 6 months or severe left ventricular failure), (b) neuromuscular disease affecting respiratory muscles, (c) moderate to severe respiratory disease or documented hypoxemia or awake SaO2 < 92% or (d) psychiatric disease that limited the ability to give informed consent. Full informed consent and baseline demographic data were obtained. Patients completed the Berlin questionnaire before randomization into either home-based management approach (Group A) or hospital-based management approach (Group B). Randomization was performed by a computerized random table into either group A) home-based management approach or group B) hospital-based management approach by a third party not involved in the trial [10].

Patients in group A underwent a level 3 validated home sleep study with the Embletta device (Embletta™ PDS) (Medcare, Iceland) [18] which is a pocket-sized, digital, multi-channel recording device that measures airflow through a nasal cannula connected to a pressure transducer, providing an AHI based on recording time. It also detects both respiratory and abdominal efforts through the effort sensor and can differentiate between obstructive and central events. Built-in position sensors are also available to differentiate supine and non supine events. Patients were instructed by nurses how to operate the device for the sleep recording and estimate their time of sleep. Respiratory events were scored when desaturations of at least 4% occurred in the absence of moving artifacts and irrespective of co-existing changes in snoring or heart rate. The Embletta™ PDS default settings for apneas and hypopneas were used in this study. An apnea was defined as a decrease in airflow by 80% of baseline for at least 10 s. A hypopnea was defined as a decrease in airflow by 50% of baseline for at least 10 s. The Embletta™ PDS AHI used for analysis was automatically analyzed by the Embletta™ PDS software which was available for reviewing and rescoring by the clinician [18].

Patients in group B underwent overnight PSG (Alice LE, Respironics, USA) at the hospital recording electroencephalogram (EEG), electro-oculogram, submental electromyogram (EMG), bilateral anterior tibial EMG, electrocardiogram, chest and abdominal wall movement by inductance plethysmography, airflow measured by a nasal pressure transducer [PTAF2, Pro-Tech, Woodinville, WA, USA] and supplemented by an oral thermister, and finger pulse oximetry as described in our previous studies [19, 20]. Apnea was defined as cessation of airflow for > 10 s with drop in the peak thermal sensor excursion by ≥90% of baseline whereas hypopnea as a reduction of nasal pressure airflow of ≥30% of baseline for > 10 s plus an oxygen desaturation of ≥4% [21]. This study was approved by the Ethics Committees of the Chinese University of Hong Kong (CREC-2011.215-T) and registered at ClinicalTrials.gov (Identifier: NCT01828216). Written informed consent was obtained from all subjects enrolled in this study.

## Statistical analysis

The results of the Berlin questionnaire were compared with the AHI obtained by PSG and the portable home monitoring by the Embletta™ PDS using sensitivity, specificity, positive predictive value (PPV) and negative predictive value (NPV) to evaluate the efficacy of the questionnaire to screen and assess severity of OSAS with different AHI cut-off at AHI > 5 h, > 15/h and > 30 h, which corresponded to mild, moderate and severe disease respectively. The comparisons were plotted graphically using receiver operating characteristic (ROC) curve analysis. The greater the area under the curve, the better the instrument would be. Data were analyzed by the Statistical Package of the Social Science (SPSS) for Windows, Version 22.0 (SPSS Inc., Chicago, IL, USA).

## Results

**Fig. 1:**
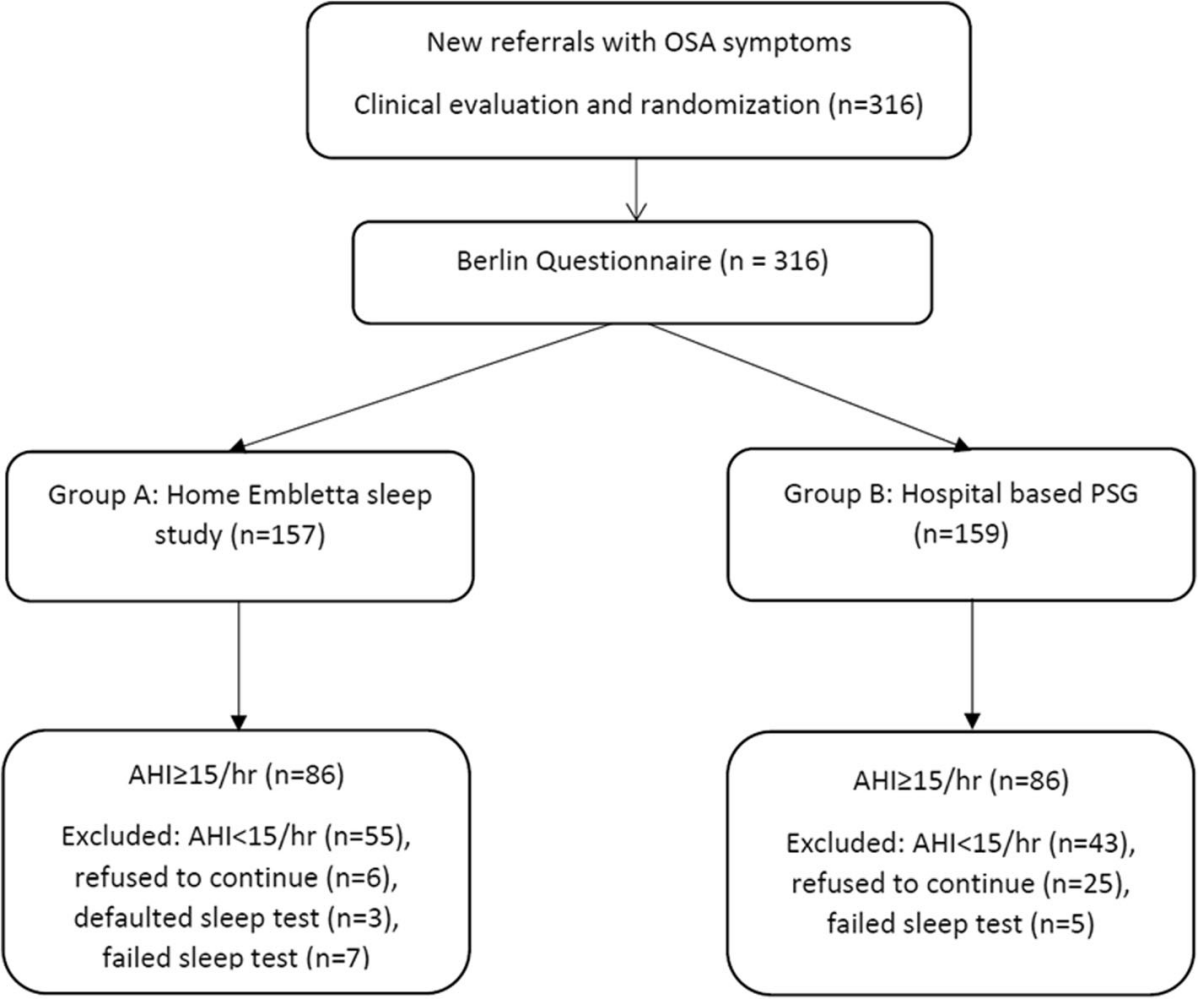
Flow chart of the study. Consort diagram of participants randomized to the home-based versus hospital-based management pathways

Altogether 316 subjects newly referred with OSAS symptoms were recruited and randomized into group A (*n* = 157) and group B (*n* = 159) as shown in Fig. 1. All of them had completed the Berlin questionnaire before sleep studies. The prevalence of moderate to severe OSAS defined as apnea-hypopnea index (AHI) ≥ 15/h was 54% (86 subjects in both group A and B). In group A, eight subjects defaulted and 7 failed the sleep tests, while 5 failed PSG and 25 refused to continue the study in group B. After exclusion of those who did not complete the sleep studies, the Berlin questionnaire identified 69.7% (*n* = 99) of subjects as high risk in group A and 77.5% (*n* = 100) in group B.. Table 1 shows the baseline characteristics between the two groups which were similar for most of the variables except for lower AHI and higher ESS in group A.

**Table 1:** Baseline characteristics between home-based (Group A) vs hospital-based approach (Group B) for diagnosis of OSAS

	Home (*n* = 157)	Hospital (*n* = 159)	*P*-value
Age	51.0 (12.9)	52.1 (11.3)	0.403
Male sex [n(%)]	107 (68.2%)	118 (74.2%)	0.264
Current smoker [n(%)]	20 (12.8%)	24 (15.1%)	0.691
Current drinker [n(%)]	26 (23.0%)	31 (26.3%)	0.765
Congestive Heart Failure [n(%)]	6 (3.8%)	5 (3.1%)	0.769
Diabetes Mellitus [n(%)]	25 (15.9%)	30 (18.9%)	0.554
Hypertension [n(%)]	72 (45.9%)	86 (54.1%)	0.177
Stroke [n(%)]	5 (3.2%)	2 (1.3%)	0.281
Ischemic Heart Disease [n(%)]	9 (5.7%)	11 (6.9%)	0.818
Hyperlipidemia [n(%)]	32 (20.4%)	32 (20.1%)	1.000
BMI (kg/m^2^)	27.4 (5.3)	28.3 (4.6)	0.115
Neck circumference (cm)	38.6 (3.6)	39.6 (3.8)	0.013
Waist circumference (cm)	95.4 (12.1)	97.3 (11.2)	0.171
Hip circumference (cm)	102.2 (9.5)	101.6 (8.1)	0.527
ESS (0–24)	11.0 (5.6)	9.6 (5.4)	0.025
AHI (events/hr)	24.1 (20.7)	30.8 (27.5)	0.023
ODI (events/hr)*	21.7 (19.8)	22.6 (23.4)	0.724
Berlin questionnaire (high risk) [n(%)]	102 (65.5%)	102 (77.3%)	0.064
Berlin questionnaire (low risk) [n(%)]	47 (31.5%)	30 (22.7%)	0.064

**Fig. 2:**
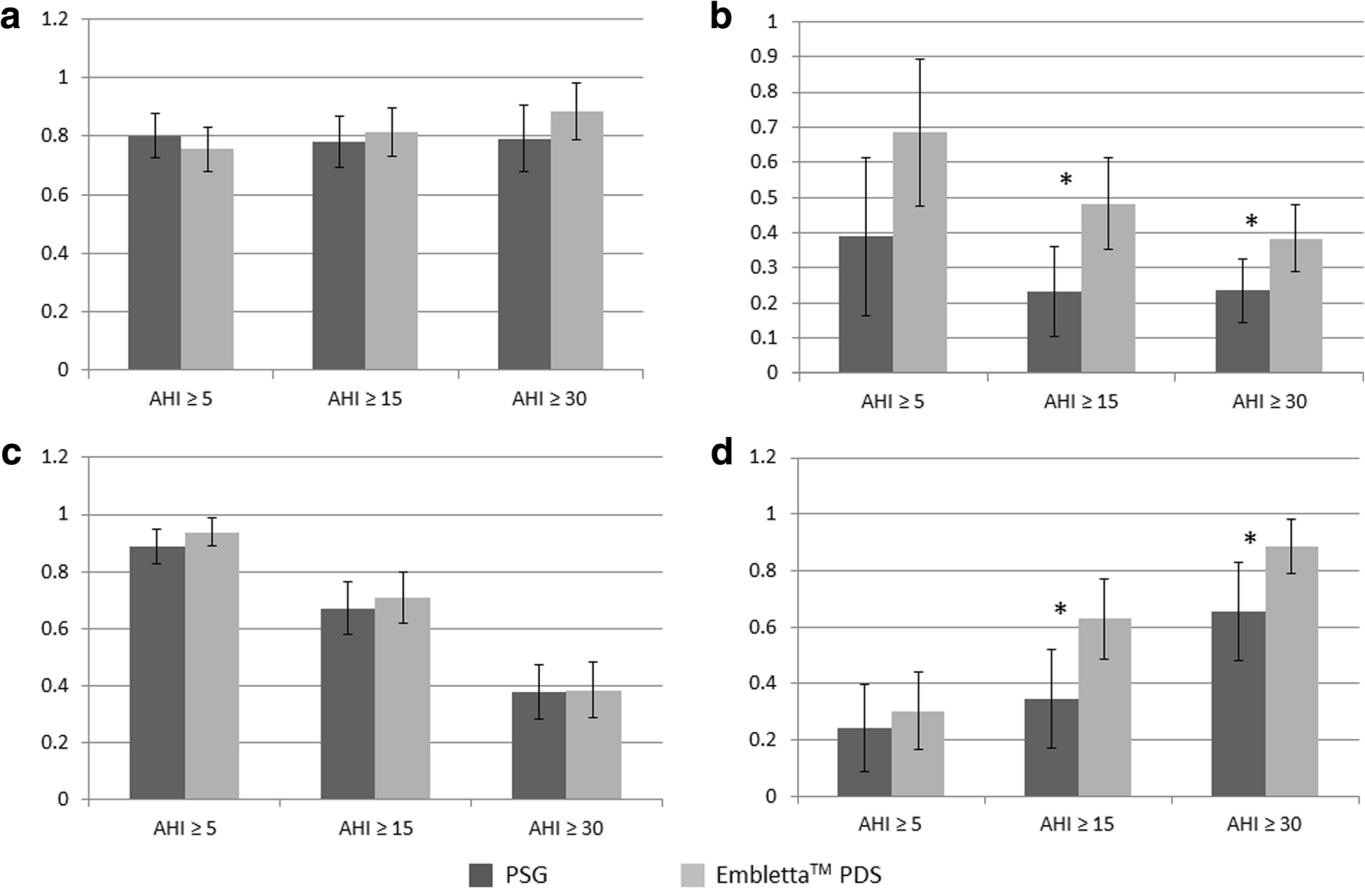
Prediction of OSAS by Berlin questionnaire againast polysomnography and home sleep test. Sensitivty (**a**), specificity (**b**), positive predictive value (**c**) and negative predictive value (d) of Berlin questionnaire in predicting the diagnosis of OSAS from polysomnography (PSG) and home sleep monitoring Embletta™ PDS. *p < 0.05*

Figure 2 shows the sensitivity, specificity, positive (PPV) and negative predictive value (NPV) of the result from Berlin questionnaire when compared against PSG and home sleep monitroing Embletta™ PDS. The sensitivity of the questionnaire was > 75% at all AHI levels among two groups, with the highest results at AHI ≥ 5/h in PSG (sensitivity 0.802) and AHI ≥ 30/h in home study group (sensitivity 0.884). The questionnaire was shown to have excellent PPV at low AHI level (0.890 in PSG and 0.939 in home study group at AHI ≥ 5/h) and NPV at high AHI level (0.655 in PSG and 0.884 in home study group at AHI ≥ 30/h). There are significant differences in the specificity of the questionnaire in comparisons to PSG and home sleep study at the AHI level of ≥15 and ≥ 30/h with higher correlation of the questionnaire with the home sleep study. The questionnaire also showed higher NPV when compared with home sleep study than with PSG at the AHI level of ≥15 and ≥ 30/h. The results of individual items including the likelihood ratios of the questionnaire when compared against PSG and AHI are shown in Table 2.

**Table 2:** Comparison of the performance of the individual items with confidence intervals in the Berlin questionnaire for prediction of OSA against polysomnography and home sleep study

	AHI ≥ 5/h		AHI ≥ 15/h		AHI ≥ 30/h	
	PSG	Embletta	PSG	Embletta	PSG	Embletta
Sensitivity	0.80 (0.73, 0.88)	0.76 (0.68, 0.83)	0.78 (0.69, 0.87)	0.81 (0.73, 0.90)	0.79 (0.68, 0.91)	0.88 (0.79, 0.98)
Specificity	0.39 (0.16, 0.61)	0.68 (0.48, 0.89)	0.23 (0.11, 0.36)	0.48 (0.35, 0.61)	0.24 (0.14, 0.33)	0.38 (0.29, 0.48)
PPV	0.89 (0.83, 0.95)	0.94 (0.89, 0.99)	0.67 (0.58, 0.76)	0.71 (0.62, 0.80)	0.38 (0.29, 0.48)	0.38 (0.29, 0.48)
NPV	0.24 (0.09, 0.40)	0.30 (0.17, 0.44)	0.35 (0.17, 0.52)	0.63 (0.48, 0.77)	0.66 (0.48, 0.83)	0.88 (0.79, 0.98)
LR+	1.31 (0.90, 1.91)	2.39 (1.23, 4.68)	1.02 (0.83, 1.24)	1.57 (1.20, 2.06)	1.03 (0.86, 1.25)	1.43 (1.19, 1.73)
LR-	0.51 (0.26, 1.02)	0.36 (0.23, 0.55)	0.95 (0.49, 1.86)	0.39 (0.23, 0.65)	0.89 (0.45, 1.75)	0.30 (0.12, 0.72)

**Fig. 3:**
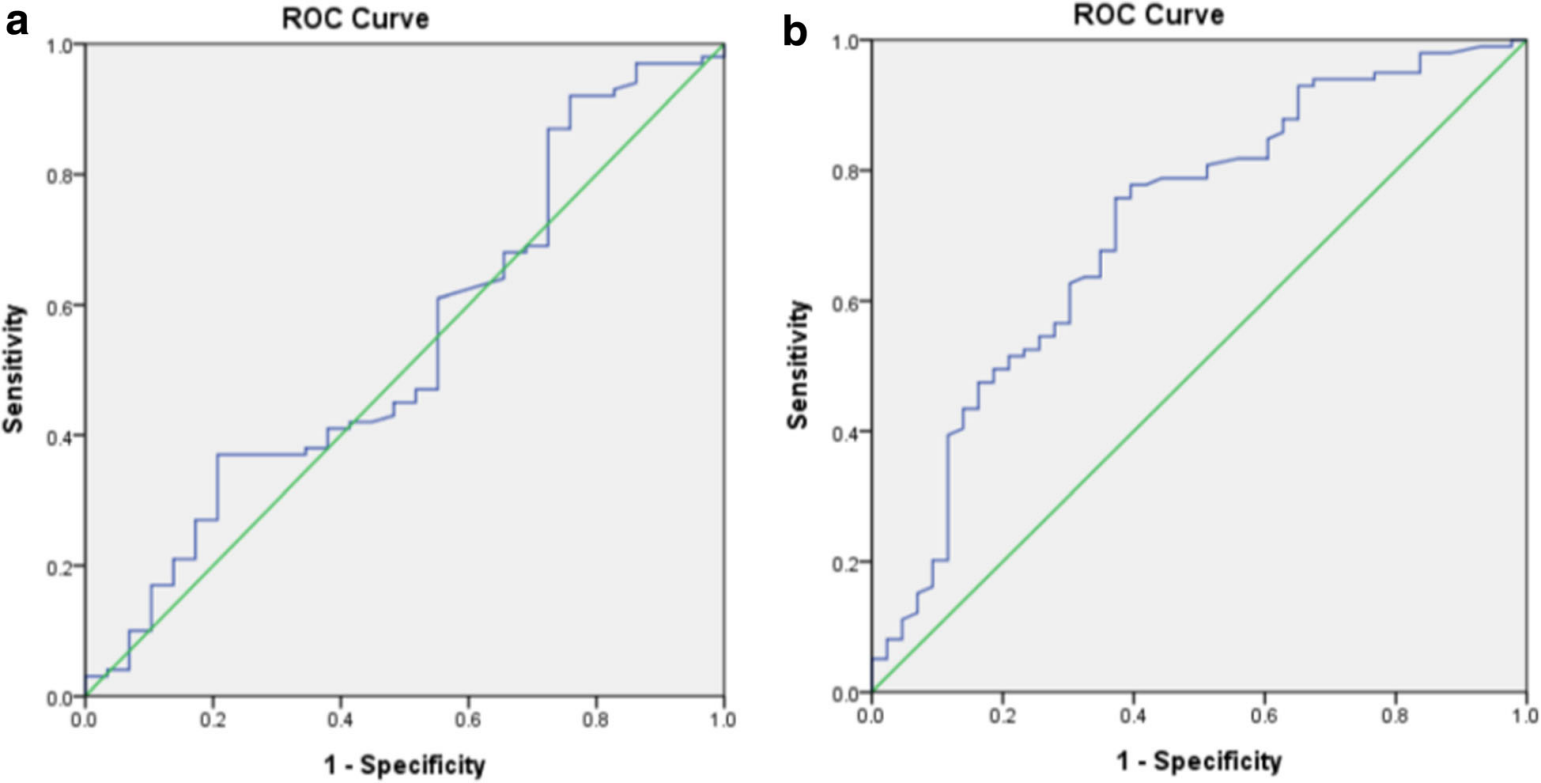
Diagnostic accuracy of the Berlin questionnaire. Reciever operative curve (ROC) analysis of the diagnostic accuracy of the Berlin questionnaire in assessing AHI based on hospital-based polysomonography (3a) and home Embletta sleep test (3b)

Further analysis of the diagnostic accuracy of the Berlin questionnaire in assessing AHI was performed with the ROC based on PSG (area under curve (AUC) = 0.539. 95%CI 0.417, 0.661) and based on home Embletta study (AUC = 0.712, 95%CI 0.617, 0.907) (Fig. 3). The questionnaire was not reliable in predicting OSAS through PSG AHI whereas there was some predictive ability in discriminating patients with OSAS from normal subjects based on home Embletta sleep test.

## Discussion

This study is the first study comparing the diagnostic accuracy of the Berlin qeuestionnaire in predicting the diagnosis of OSAS determined by PSG and home sleep test. Though the questionnaire has shown a high sensitivity in screening for the disease, it has poor specificity in the diagnosis. The Berlin questionnaire is a commonly used questionnaire in epidemiological and clinical research with variable sensitivity and specificity in different studies [22–26]. One of the reasons for the variability is related to the method in the validation. With the growing literature in support of the ambulatory approach as an alternative strategy in managing patients with a high clinical probability of OSAS, home-based management approach is shown to be more cost effective with shorter waiting time and substantial cost savings in the diagnosis of OSAS when compared with inhospital PSG [10]. This Embletta™ PDS has been validated in various studies [18, 27] against simultaneous PSG with high sensitivity and specificity. As the device is a type III monitor without measurement of EEG, total recording time was used as the denominator in calcuating the AHI, which could be underestimated if the sleep quality was not good. This is in concordance with the higher specificity and NPV of the questionnaire against Embletta™ PDS when compared with that against PSG. Recently, Tan et al validated the use of the Berlin qestionnaire in predicting OSAS as diagnosed by the Embletta device with high specificity throughout different AHI [26]. In contrast to our findings, the sensitivity of the questionnaire was 58.8% in predicting an AHI ≥15/h while it rose to 76.9% in predicting severe OSAS. Thus the use of questionnaire is useful only in predicting patients with severe OSAS. The latest meta-analysis with review on the questionnaire’s diagnostic utility against PSG showed similarly good sensitivity for detecting clinically relevant OSAS in the sleep clinic population, but low specificity in screening the general populations [28]. Nevertheless, the high PPV of the questionnaire in those patients having AHI > 5/h in may help selecting patients with suspected disease to have fast track investigation, especially among those with cardiovascular comorbidites.

While the Berlin Quesionnaire categorizes patients into high or low risk for OSAS according to symptoms and body mass index (BMI), STOP-Bang questionnaire is another validated screening tool with four subjective (STOP: Snoring, Tiredness, Observed apnea and high blood Pressure) and four demographic items (Bang: BMI, Age, Neck circumference, Gender) [15]. This questionnaire has been widely used in preoperative clinics [15, 29], sleep clinics [30, 31] and the general populations [32] for predicting patients at high risk of OSAS. A meta-analysis showed that the probability of moderate and severe OSAS steadily increased with a higher STOP-Bang score in patients at high risk of OSAS [33]. However, the evidence of its use in general population is not known. The US Preventive Services Task Force (USPSTF) has commented that none of the potential screening questionnaires and clinical prediction tools including ESS, STOP questionnaire, STOP-Bang questionnaires, the Berlin Questionnaire, Wisconsin Sleep Questionnaire, and the Multivariable Apnea Prediction (MVAP) tool have been adequately validated in a primary care setting [34]. The American Academy of Sleep Medicine has also made a strong recommendation that clinical tools, questionnaires and prediction algorithms not be used to diagnose OSA in adults, in the absence of polysomnography or home sleep apnea testing [35].

Our study has several limitations. Firstly, this was an ancillary study from our previous study with sample size originally being powered to demonstrate non-inferiority of the ambulatory approach versus the hospital based approach with respect to change in ESS, the primary outcome measure [10]. Nevertheless, according to the minimum sample size for sensitivity and specificity reported in Bujang and Adnan [36], our current sample size should be enough. Secondly, the baseline characteristic between the two groups were not matched in which increasing the sample size could be helpful to eliminate the difference in the future studies. Thirdly, the Berlin questionnaire was developed as a screening tool for OSAS in unselected, primary care populations while the population we recruited was sleepy and symptomatic patients with ESS > 9 and at least 2 OSAS related symptoms. The lower diagnostic performance might have been related to exclusion of non-sleepy patients with OSAS. The difference in the studied population may have altered the performance characteristics of the questionnaire. While expecting the Berlin questionnaire to perofrm even better in sleepy patients, our study results might be a conservative estimate of unreliability. Fourthly, different scoring apnea/hypopnea rules for Embletta PDS and PSG were applied in the study as in prevoius validation study [18], the difference in the scoring rules between the two systems together with the limitation of measuring total recording time instead of actual sleep time could be the potential bias in the difference in AHI.

## Conclusion

The Berlin Questionanire was unreliable in our patient population in predicting OSAS by PSG-AHI whereas the ability to differentiate patients with OSAS from normal was better with home Embletta-AHI.

## Abbreviations

AHI: Apnea-hypopnea index
AUC: Area under curve
BMI: Body mass index
CPAP: Continuous positive airway pressure
EEG: Electroenchelogram
Embletta PDS: Embletta™ portable diagnostic system
ESS: Epworth Sleepiness Score
HK: Hong Kong
NPV: Negative predictive value
OSAS: Obstructive sleep apnea syndrome
PPV: Positive predictive value
PSG: Polysomnography
RCT: Randomized controlled trial
ROC: Receiver operating characteristic
SPSS: The Statistical Package of the Social Science
